# Minimally Invasive Extirpation of an Eden Type II Dumbbell-Shaped Mediastinal Tumor Using a Posterior and Uniportal Thoracoscopic Approach without Changing the Patient’s Position: A Case Report

**DOI:** 10.70352/scrj.cr.25-0763

**Published:** 2026-03-20

**Authors:** Takao Ishimura, Yoshifumi Sano, Seiji Shigekawa, Nozomi Takahashi, Masashi Takeda, Takahito Sugihara, Yosuke Kiriyama, Yu Mori, Nobuhiko Sakao, Shinji Otani, Hironori Izutani

**Affiliations:** 1 Department of Cardiovascular and Thoracic Surgery, Ehime University Graduate School of Medicine, Toon, Ehime, Japan; 2 Department of Thoracic Surgery, Minami-matsuyama Hospital, Matsuyama, Ehime, Japan; 3 Department of Neurosurgery, Ehime University Graduate School of Medicine, Toon, Ehime, Japan

**Keywords:** mediastinal tumor, dumbbell-shaped tumor, uniportal video-assisted thoracic surgery, prone position

## Abstract

**INTRODUCTION:**

Dumbbell-shaped posterior mediastinal tumors, most commonly schwannomas, sometimes extend through the intervertebral foramen into the spinal canal and often require a combined posterior and thoracic approach. Although complete resection is essential, reducing surgical invasiveness remains an important clinical priority. We report a case of an Eden type II dumbbell-shaped schwannoma that was successfully resected using a minimally invasive single-stage approach combining posterior surgery and uniportal thoracoscopic surgery, performed entirely in the prone position without intraoperative repositioning.

**CASE PRESENTATION:**

A 55-year-old man presented with intermittent anterior chest and back pain. Imaging revealed a 20-mm, dumbbell-shaped tumor at the left T9 intervertebral foramen with suspected intradural extension. A single-stage combined posterior and thoracoscopic resection was performed without intraoperative repositioning. Through an 8-cm posterior incision, hemilaminectomy, dural opening, internal decompression, and nerve root transection were conducted, allowing for mobilization of the tumor. Subsequently, a 3-cm uniportal thoracoscopic approach facilitated complete tumor extraction from the mediastinum. The tumor was removed en bloc, and pathology confirmed a benign schwannoma. The postoperative course was uneventful, and the patient was discharged on POD 4. At 6 months, he remained asymptomatic except for mild intercostal numbness, with no radiologic evidence of recurrence.

**CONCLUSIONS:**

This case demonstrates the feasibility and efficacy of a minimally invasive technique for Eden type II dumbbell-shaped schwannoma, combining posterior and uniportal thoracoscopic approaches without patient repositioning. This approach reduced surgical trauma, shortened operative and recovery times, and offered excellent cosmetic and clinical outcomes, which may serve as a valuable option for selected patients requiring resection of dumbbell-type mediastinal tumors.

## Abbreviations

RATS
: robot-assisted thoracic surgery
VATS
: video-assisted thoracoscopic surgery

## INTRODUCTION

A dumbbell-shaped mediastinal tumor refers to a tumor characterized by a shape resembling a dumbbell, typically involving the mediastinum and extending into adjacent structures such as the spinal canal. Dumbbell-shaped posterior mediastinal tumors are most commonly schwannomas, most of which are benign.^1)^ Surgical treatment often requires a dual approach involving both dorsal and thoracic access.^2)^ However, from an oncological perspective, a less invasive approach is highly desirable. Here, we report a case of a dumbbell-shaped schwannoma with both intra- and extradural extension successfully treated using a minimally invasive technique. The procedure involved hemilaminectomy, dural incision, and nerve root resection through an 8-cm posterior incision, followed by uniportal thoracoscopic tumor resection via a 3-cm left thoracic incision. Remarkably, the entire surgery was performed in the prone position without the need for repositioning, demonstrating a minimally invasive surgical approach.

## CASE PRESENTATION

A 55-year-old man had occasionally experienced pressure-like pain from the anterior chest to the back for approximately 10 years, which spontaneously subsided without medical attention. However, about 4 months ago, he experienced the same pressure-like pain in the anterior chest and back and presented to another hospital. During the evaluation, a posterior mediastinal tumor extending into the intervertebral foramen was identified, and he was subsequently referred to our department.

Past medical history: Stroke, hypertension, hyperlipidemia, and type 2 diabetes mellitus

Smoking history: 16.5 pack/years

Occupational history: Worked in manufacturing

**Fig. 1:**
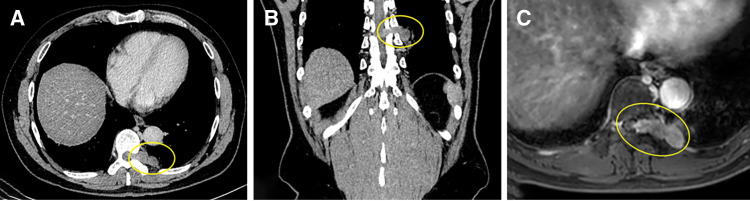
(**A**) Axial and (**B**) coronal contrast-enhanced CT identified a 20-mm dumbbell-shaped mass protruding from the left T9 intervertebral foramen into the spinal canal. (**C**) MRI revealed a dumbbell-shaped tumor originating from the left T9 intervertebral foramen, suggesting possible extension into the intradural space (yellow circles indicate the tumor).

Contrast-enhanced CT revealed a 20-mm dumbbell-shaped tumor extending from the left T9 intervertebral foramen into the spinal canal (**Fig. 1A** and **1B**). MRI demonstrated a dumbbell-shaped tumor extending from the T9 intervertebral foramen to the left, suggesting possible extension into the intradural space (**Fig. 1C**).

Based on the preoperative diagnosis, a dumbbell-shaped posterior mediastinal schwannoma extending both inside and outside the spinal canal (Eden type II) was strongly suspected, and a combined posterior and transthoracic approach was deemed necessary for surgical resection.

### Surgical procedure

**Fig. 2:**
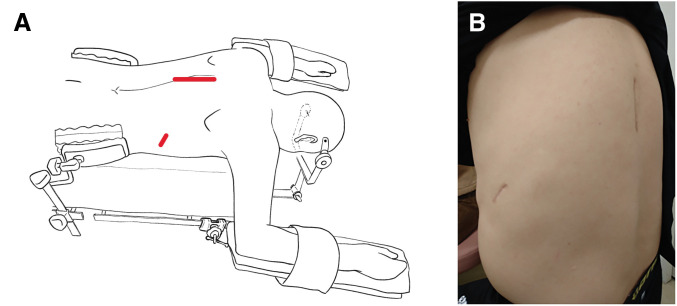
(**A**) Schematic illustration of the prone position used during surgery, including arm positioning and body support. The red line indicates the skin incision line. (**B**) The procedure was completed using only 2 incisions.

The surgery was performed entirely in the prone position without repositioning the patient. Oral endotracheal intubation was conducted using a Broncho-Cath Endobronchial Tube (Medtronic, Minneapolis, MN, USA). Both upper extremities were elevated and positioned to avoid excessive abduction. Pelvic supports were applied to accommodate potential table rotation during the procedure. The head was secured using a pin fixation system to allow intraoperative confirmation and adjustment of the endotracheal tube (**Fig. 2A** and **2B**). Based on the preoperative radiological assessment indicating that the tumor was predominantly extradural and that significant spinal cord manipulation was unlikely, intraoperative neurophysiological monitoring was not employed.

### Posterior approach

The spinal surgeons began the procedure with a posterior approach. A midline skin incision approximately 8 cm in length was made on the back. The left hemilamina of T9 was resected without removing the transverse process, exposing a well-demarcated tumor extending laterally. The tumor appeared dark red, encapsulated, and continuous with the dura mater along the nerve root. Upon opening the dura, the tumor was found to extend from the extradural to the intradural space.

Intraoperative rapid pathological diagnosis confirmed the tumor to be a schwannoma. Using instruments including the CUSA Clarity (Integra Japan, Tokyo, Japan), internal decompression of the tumor was performed. The T9 nerve root was identified, transected at its root, and the tumor was mobilized into the extradural space. A dural defect created at the site of continuity between the extradural and intradural portions was repaired by placing artificial dura mater (DuraGen; Integra Japan) both inside and outside the dura, and was reinforced with fibrin glue. After partial dissection of the extradural tumor from surrounding tissues, the incision was closed, and the procedure was transitioned to a thoracoscopic approach by the thoracic surgeons.

### Thoracoscopic approach

**Fig. 3:**
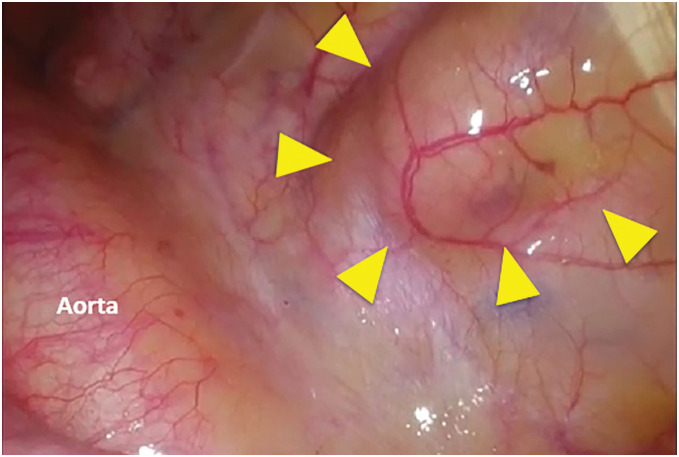
Intraoperative findings. The tumor appeared as a protrusion of the mediastinal pleura adjacent to the descending aorta.

A 3-cm skin incision was made along the mid-axillary line at the 8th intercostal space while the patient remained in the prone position. The tumor was identified near the 9th rib as an elevation of the mediastinal pleura on the dorsal side of the descending aorta (**Fig. 3**). The mediastinal pleura was incised along the lateral margin of the elevation using electrocautery, and the tumor was dissected sharply with electrocautery and bluntly as needed, with ligaments and fibrous bands severed using the LigaSure Vessel Sealing System (Medtronic). Preoperative imaging did not clearly identify the artery of Adamkiewicz in this case. Intraoperatively, no major intercostal arteries were found to be directly involved with the tumor, allowing safe tumor mobilization and resection without vascular injury.

Communication with the surgical field from the posterior approach was achieved, allowing the tumor to be mobilized and extracted through the intervertebral foramen. Tumor resection was completed. A 20-Fr silicone thoracic tube was inserted into the thoracic cavity through the incision, without placement of a dorsal drainage tube, and the operation was completed.

### Pathological findings

**Fig. 4:**
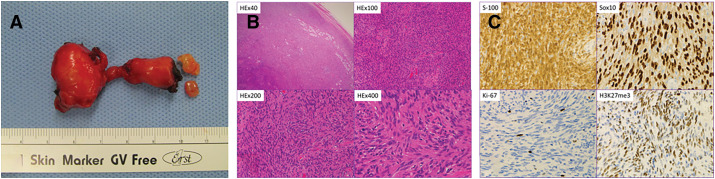
(**A**) Microscopic findings of the surgically resected tumor. En bloc resection of the dumbbell-shaped tumor was achieved. (**B**) Histological examination revealed densely packed spindle cells, indicative of a schwannoma. (**C**) Immunostaining supported the diagnosis of benign schwannoma.

The resected tumor measured 4 cm macroscopically and comprised both intrathoracic and intraspinal components, which were removed en bloc. Histologically, hematoxylin and eosin staining revealed a dense proliferation of spindle-shaped cells. Immunohistochemical analysis showed positivity for S-100 and SOX10, supporting a diagnosis of schwannoma. The presence of H3K27me3-positive tumor cells and a low Ki-67 labeling index of approximately 1% further supported the benign nature of the tumor (**Fig. 4A**–**4C**).

### Postoperative course

On the evening of POD 1, the patient exhibited a slight decrease in the consciousness level. However, there were no significant changes in motor or sensory function, and blood glucose levels, electrolytes, and other laboratory findings were unremarkable. Emergency non-contrast head and chest CT and brain MRI were performed, which revealed no intracranial abnormalities or other acute findings. Although alterations in cerebrospinal fluid pressure following dural opening and repair were considered, there were no clinical findings suggestive of low- or high-pressure cerebrospinal fluid syndrome, such as postural headache or vomiting. By the morning of POD 2, the patient’s condition had improved, and the chest tube was removed. The patient was discharged on POD 4 with independent ambulation.

At 6 months postoperatively, the patient remained asymptomatic except for numbness in the 9th intercostal nerve region, with no abnormalities detected on MRI, indicating a favorable clinical course.

## DISCUSSION

Dumbbell-shaped tumors were first described by Antoni in 1920 based on their morphological characteristics, with Heuer providing the first systematic report in 1929.^3)^ The classification of dumbbell tumors is commonly based on the morphological criteria proposed by Eden, consisting of (1) intra- and extradural type, (2) intra- and extradural with paravertebral extension, (3) extradural with paravertebral extension, and (4) foraminal and paravertebral type.^4)^ Among these, type II and type III, in which the tumor extends from the spinal canal through the intervertebral foramen into the extradural space, are considered true dumbbell-type tumors. Based on preoperative radiological assessment, the tumor was located in both the paravertebral region and the spinal canal, connected through the intervertebral foramen, suggesting an Eden type II or type III classification. Intraoperative findings confirmed that the tumor extended from the paravertebral region into both the intradural and extradural spaces, leading to a final diagnosis of an Eden type II dumbbell tumor.

Approximately 30%–40% of thoracic dumbbell tumors are asymptomatic at diagnosis.^4)^ As the tumor enlarges, it may cause local compression of adjacent structures or involve the spinal canal, necessitating therapeutic intervention.^1)^ The majority of dumbbell tumors are benign, including schwannomas and neurofibromas, accounting for approximately 90%.^2)^ However, differentiating between benign and malignant tumors using imaging alone is challenging,^5)^ making surgical resection the first-line treatment for both definitive diagnosis and management. Complete tumor resection is crucial to prevent recurrence. However, given the predominantly benign nature of these tumors, achieving radical resection while minimizing surgical invasiveness is essential.

Surgical approaches for dumbbell tumors include (1) a posterior approach via laminectomy, (2) a thoracic approach via thoracotomy or thoracoscopy, and (3) a combined approach. In cases of Eden type II posterior mediastinal dumbbell tumors, such as in this report, tumor morphology often makes resection via a single posterior or thoracic approach challenging and potentially risky. In the present case, the paravertebral component extended into the posterior mediastinum adjacent to the descending aorta. Consequently, complete and safe resection was considered technically challenging with either a posterior approach or a thoracoscopic approach alone; therefore, a combined approach was adopted.

Historically, the posterior and lateral thoracic approaches were performed in a staged manner.^6)^ However, to reduce the invasiveness associated with multiple procedures, single-stage surgery has gained increasing favor. Additionally, in thoracic approaches, there has been a shift from open thoracotomy to video-assisted thoracoscopic surgery (VATS) to minimize surgical invasiveness further.^7,8)^ Recently, uniportal VATS has been reported as a feasible surgical option. Our group previously reported a similar case treated with a combined posterior and uniportal thoracoscopic approach; however, intraoperative repositioning of the patient was required in that procedure.^9)^ Even in single-stage procedures, traditional methods have involved positioning the patient in the prone position for the posterior approach, followed by repositioning to the lateral decubitus position for VATS. However, Okazaki et al.^10)^ reported a technique in which robot-assisted thoracic surgery (RATS) was performed via the lateral approach while maintaining the patient in the prone position, thus eliminating the need for intraoperative repositioning. However, this technique still required 4 incisions in the lateral thoracic region.

To further enhance minimally invasive techniques, we successfully performed a complete resection of an Eden type II dumbbell–type schwannoma using only 2 incisions: a midline posterior incision (8 cm) and a uniportal thoracoscopic incision (3 cm), without repositioning the patient. This approach represents a minimally invasive surgical technique, combining a single-stage procedure without intraoperative repositioning with only 2 small incisions. Performing both the posterior and thoracoscopic procedures entirely in the prone position offers several important advantages. Gravitational displacement of the lung reduces lung compression, facilitating intrathoracic manipulation. In addition, the prone position minimizes the risk of inadvertent contact between surgical instruments and the descending aorta, as well as provides an improved and stable visual field of the posterior mediastinum. Furthermore, eliminating intraoperative repositioning shortens operative time, reduces hemodynamic and respiratory fluctuations, and decreases the risk of accidental dislodgement of devices such as the endotracheal tube and intravenous lines. These features suggest that this approach may be a safe and effective surgical option for selected patients with dumbbell-shaped mediastinal tumors requiring combined posterior and thoracic approaches.

## CONCLUSIONS

We successfully performed a minimally invasive surgical procedure for an Eden type II dumbbell-shaped schwannoma by combining a single posterior approach with uniportal thoracoscopic surgery, without the need for intraoperative repositioning. This approach yielded excellent outcomes. We hope that this technique will become more widely adopted in the future.

## DECLARATIONS

### Availability of data and materials

The data supporting the conclusions of this article are included within this report.
